# Irreversible electroporation (IRE) in renal cell carcinoma (RCC): a mid-term clinical experience

**DOI:** 10.1007/s00330-021-07846-5

**Published:** 2021-03-30

**Authors:** Tze Min Wah, James Lenton, Jonathan Smith, Paul Bassett, Satinder Jagdev, Christy Ralph, Naveen Vasudev, Selina Bhattarai, Michael Kimuli, Jon Cartledge

**Affiliations:** 1 Division of Diagnostic and Interventional Radiology, Institute of Oncology, St. James’s University Hospital, Beckett Street, Leeds, LS9 7TF UK; 2 Statsconsultancy Ltd., 40 Longwood Lane, Amersham, Bucks HP7 9EN UK; 3 Division of Medical Oncology, Institute of Oncology, St. James’s University Hospital, Beckett Street, Leeds, LS9 7TF UK; 4 Division of Pathology, Institute of Oncology, St. James’s University Hospital, Beckett Street, Leeds, LS9 7TF UK; 5 Division of Urology, Institute of Oncology, St. James’s University Hospital, Beckett Street, Leeds, LS9 7TF UK

**Keywords:** Electroporation, Renal cancer, Safety, Complication, Survival rates

## Abstract

**Objectives:**

To evaluate the safety and efficacy of CT-guided IRE of clinical T1a (cT1a) renal tumours close to vital structures and to assess factors that may influence the technical success and early oncological durability.

**Methods:**

CT-guided IRE (2015–2020) was prospectively evaluated. Patients’ demographics, technical details/success, Clavien-Dindo (CD) classification of complications (I–V) and oncological outcome were collated. Statistical analysis was performed to determine variables associated with complications. The overall 2- and 3-year cancer-specific (CS), local recurrence-free (LRF) and metastasis-free (MF) survival rates are presented using the Kaplan-Meier curves.

**Results:**

Thirty cT1a RCCs (biopsy-proven/known VHL disease) in 26 patients (age 32–81 years) were treated with IRE. The mean tumour size was 2.5 cm and the median follow-up was 37 months. The primary technical success rate was 73.3%, where 22 RCCs were completely IRE ablated. Seven residual diseases were successfully ablated with cryoablation, achieving an overall technical success rate of 97%. One patient did not have repeat treatment as he died from unexpected stroke at 4-month post-IRE. One patient had CD-III complication with a proximal ureteric injury. Five patients developed > 25% reduction of eGFR immediately post-IRE. All patients have preservation of renal function without the requirement for renal dialysis. The overall 2- and 3-year CS, LRF and MF survival rates are 89%, 96%, 91% and 87%.

**Conclusion:**

CT-guided IRE in cT1a RCC is safe with acceptable complications. The primary technical success rate was suboptimal due to the early operator’s learning curve, and long-term follow-up is required to validate the IRE oncological durability.

**Key Points:**

*• Irreversible electroporation should only be considered when surgery or image-guided thermal ablation is not an option for small renal cancer.*

*• This non-thermal technique is safe in the treatment of small renal cancer and the primary technical success rate was 73.3%.*

*• This can be used when renal cancer is close to important structure.*

## Introduction

Since 2017, both AUA and ESMO have issued guidance where image-guided thermal ablation can be considered a valid treatment option for small renal cancer (< 3 cm) [1, 2]. It is however well recognised that thermal ablation with radiofrequency ablation or cryoablation can cause significant collateral damage to the vital structures in proximity especially renovascular pedicles or ureter [3, 4], hence the need for various protective techniques to minimise injury [5, 6]. Irreversible electroporation (IRE) is the novel non-thermal ablative technology, where it delivers high-voltage electrical pulses to cause irreversible nanopores in the cellular membrane leading to apoptosis [7, 8]. The laboratory evidence had shown preservation of collagen i.e. phospholipid bilayer structure where bile duct/vessels are preserved adjacent to the zone of ablation [9]. This technology has since been explored in treating renal cancer in the need of minimising collateral damage when sited in an awkward location as a problem-solving technique and the intention is not to replace the current conventional thermal ablative energy in the treatment of small renal cancer. In 2011, Pech et al had performed the first in man IRE treatment of RCC [10]. Since 2015, following approval of a new interventional procedure in our regional cancer institution, we had successfully performed the first in the country IRE treatment for a patient with central RCC to ensure nephron-sparing [11]. Over the next 5 years, we prospectively collated all patients’ clinical outcomes that were referred to our cancer institution for image-guided ablation; following consensus from the renal cancer multidisciplinary (MDT) team, IRE treatment has been performed when other thermal ablative technology had been considered unsuitable because of the risk of collateral damage. To our knowledge, this early clinical experience has the longest median follow-up period post-IRE of biopsy-proven RCC published to date. This study aims to evaluate the initial clinical safety and efficacy of IRE in the treatment of sporadic clinical T1a (cT1a) biopsy-proven RCC close to vital structures and to assess the factors that may influence the technical success and the early oncological durability.

## Materials and methods

Formal ethical approval was waived for retrospective reviewing of collated data as part of the prospective clinical registry in accordance with the Institutional Health Research Authority Framework.

### Patient selection

All patients were referred through our local urology multidisciplinary team (MDT) meeting at a regional cancer centre with an Interventional Oncology (IO) program. This is an active IO program that has provided cancer treatment for cT1a RCC since 2004 with radiofrequency ablation and cryoablation. In 2015, following formal institutional approval to use this innovative non-thermal IRE technology to treat selected cT1a RCCs due to tumour location and risk to vital structures, a small cohort of patients were offered IRE treatment following consensus at the urology MDT. The inclusion criteria were cTa RCC (< 4 cm) and the distance to vital structure, e.g. ureter, colon, solid organ and vascular pedicles, was < 1mm. The exclusion criteria were patients without the mental capacity to consent and the cT1a RCC can be treated safely with conventional thermal ablation. All patient data was prospectively recorded in the local institutional data registry from May 2015 to May 2020.

### Procedure

Prior to IRE treatment, 24 patients had biopsy-proven RCC and in 2 patients, with known VHL disease and numerous previously proven RCCs, biopsy was deemed unnecessary. All IRE ablations were performed by the most senior interventional radiologist in the IO program to ensure efficacy and safety during the initial evaluation phase (T.M.W. with 17 years of experience in the treatment of cT1 RCC with ablative technology) with the support of the IO team and the first renal case was supported by national IRE expert in liver and pancreas (P.L.) [11].

The IRE procedures were performed with the NanoKnife® IRE System and Nanoknife monopolar probes (15 cm, 19G). All the electrodes were inserted under computed tomography (CT) guidance and the needle placement planned accordingly to ensure optimal distance (between 1.5 and 2 cm) for the pairing of the IRE electrodes with an exposure tip length of 2 cm. The patients were under general anaesthesia with deep neuromuscular blockade to ensure total muscular paralysis during IRE treatment. The number of IRE electrodes used is based on the size, location and configuration of the tumour with the aim to ensure full coverage of the tumour, as the electrodes are usually sited at the periphery to bracket the tumour in its entirety.

After CT verification of the IRE electrode placement and measurement of the distance between the pairing of the electrodes, a test run of 20 pulses per electrode pair was routinely performed to determine the electrical conductivity across all the electrodes was performed. The interelectrode voltage adjustment was made based on the evaluation of post-IRE test run graphs, to ensure the current delivered was between 20 and 40 A. With optimal voltage adjustment, all the RCCs were treated with the designated voltage which was delivered for 90 pulses per electrode pair with cardiac gated during the pulsation (pulsed for 100 ms at 1 Hz). At the end of the IRE treatment, a non-enhanced CT was performed to assess the zone of ablation and to detect any immediate post treatment complication.

All patients were monitored overnight post-IRE treatment and admitted under IO program with combined care from nephrology, urology and renal oncology teams at our regional cancer centre.

### Follow-up

**Fig. 1:**
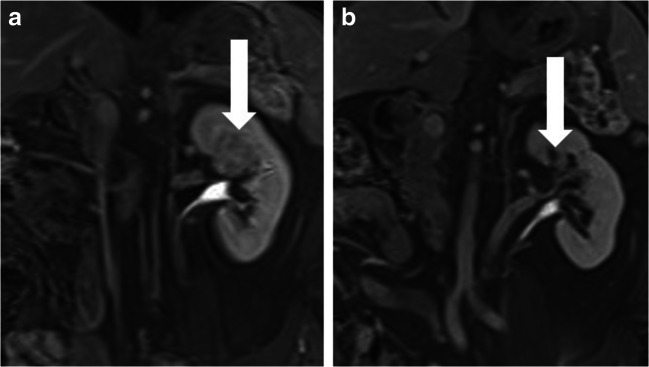
Coronal 12-min excretory phase post contrast-enhanced MRI showed a centrally located 3.5-cm biopsy-proven grade 1 eosinophilic RCC (vertical arrowed) pre-IRE (**a**) and complete resolution of tumour (vertical arrow) at 12 months post-IRE (**b**)

Imaging and clinical follow-up post-IRE were as per the schedule employed in patients after image-guided thermal ablation with radiofrequency ablation (RFA) or cryoablation (CRYO) at our regional cancer centre [4]. The protocol includes baseline MRI, imaging 1 and 3 months post treatment with MRI and 6 and 12 months post treatment with CT or MRI (Fig. 1 a and b) for those with renal impairment or iodine contrast allergy in the first year. After that, all patients are monitored annually with full-body CT to assess the thorax, abdomen and pelvis for up to 10 years. All images are read by one of the interventional radiologists in the IO team (T.M.W., J.T.S. or J.L.). The reporting standard of the treatment efficacy [12] was defined as a complete treatment response with no evidence of residual enhancing tumour and the zone of ablation encompassed the entire tumour by 3 months. Local recurrent disease was defined as any enhancement (nodular or crescentic) within the ablation zone during the follow-up imaging. The complications were collated prospectively in the registry and grade as in accordance with the Clavien-Dindo classification; major complications were defined when grades >/= III [13]. The renal functions measured by glomerular filtration rate (GFR) were monitored at baseline and 1 day post-IRE, and those with > 25% decrease from the baseline post treatment were monitored to stability by community general practitioners or nephrologists. The patients’ clinical follow-up was prospectively collated to include the overall survival (OS), cancer-specific survival (CS), local recurrence-free survival (LRFS) and metastasis-free survival (MFS) rates.

### Data analysis and statistics

Statistical analysis was performed using Stata (Stata: release 15.1. StataCorp: StataCorp LP) according to a predefined statistical analysis outline. Descriptive statistics (e.g. mean, SD and variance) were reported. Univariate logistic regression analysis was performed to determine any association between primary technical success rate, major complications (Clavien-Dindo >/= 3) and the change of pre and post treatment GFR (% GFR change) > 25% with the patients’ demographics (age and sex), nephrometry score, grade and histological type of RCC, tumour size, length of procedure and number of IRE electrodes (Table 2). Kaplan-Meier curves were used to determine the 2- and 3- year overall survival (OS), cancer-specific (CS), local recurrence-free survival (LRFS) and metastasis-free survival (MFS) and these were also documented. *p* values of less than 0.05 were considered to be statistically significant.

## Results

From May 2015 to 2020, a total of 30 cT1a renal tumours were treated with IRE under CT guidance with the intention to cure. Patient demographics, tumour characteristics, IRE procedural time/electrodes used and histology are shown in Table 1. The mean tumour size was 2.5 cm (SD+/− 9.23) and the mean R.E.N.A.L. nephrometry score of 6 (SD +/−1.97). The mean IRE intraprocedural time was 72 min (SD +/−24.2 min). The histology of the IRE-treated RCC was clear cell RCC (*n = *19; 63%), papillary RCC (*n* = 5; 16%), eosinophilic RCC (*n* = 2; 7%), chromophobe RCC (*n* = 2; 7%) and no biopsy with history of clear cell RCC in VHL disease patients (*n* = 2; 7%). Amongst the RCCs treated, 97% (29/30) were close to vital structures. The mean minimum distance was 0.2 mm (SD+/− 0.31mm), with the vital structures in close proximity being colon (*n* = 11), ureter (*n* = 11) and renovascular pedicles (*n = *7). One of the two renal tumours in the same patient was not in close proximity with any vital structure. Following IRE treatment, all the patients are monitored overnight with a mean hospital night stay of 2 (SD+/−1.96). The mean post-IRE % change in GFR was −13 ml/min/1.73 m^2^ (SD+/−16.3 ml/min/1.73 m^2^) amongst all patients.

**Table 1:** Patients’ demographic and tumour characteristics treated with IRE (2015–2020)

Parameter	IRE-treated population (*n* = 26)
Age	
Mean +/- SD	65 (+/-11.4)
Median and range	67 (32–81)
Sex	Male (17) female (9)
Tumour characteristics	(*n* = 30)
Mean +/- SD (cm)	2.5 +/- 0.93
Median and range (cm)	2.5 (1–4)
Size < 2 cm	7 (23%)
Size > or = 2 cm	23 (77%)
R.E.N.A.L. score, median (SD)	6 (1.97)
No. left vs. right (%)	15 (50%) vs 15 (50%)
Tumour polarity	
Upper (%)	4 (13.3%)
Interpolar (%)	9 (30%)
Lower (%)	17 (57%)
IRE treatment time (min), median (SD)	80 (24.2)
No. of probes, mean (SD)	5 (1.37)
Histopathology	
Clear cell RCC (%)	19 (63%)
Papillary RCC (%)	5 (16%)
Chromophobe RCC (%)	2 (7%)
Eosinophilic RCC (%)	2 (7%)
VHL with history biopsied proven clear cell RCC	2 (7%)
GFR (ml/min/1.73m^2^)	
Pre-IRE, mean (SD)	68 (17)
Post-IRE, mean (SD)	60 (19)
% change, mean (SD)	- 13 (16)

### Treatment efficacy, complications and change in eGFR

Initial technical success was achieved after the first IRE in 22 of 30 tumours at first follow-up imaging (73%; 95% confidence interval [CI]: 54%, 88%). Seven out of the 8 cases with residual disease were successfully ablated with salvage cryoablation (CRYO) at a mean period of 3 months (SD+/−1.6) from the first IRE, achieving an overall technical success rate of 97% (95% confidence interval [CI]: 83%, 100%). Salvage CRYO was the thermal energy of choice because this is the current conventional approach at the cancer institution. All the seven patients were consented regarding the high risk of permanent dialysis and the need for surgery when salvage CRYO failed. One patient did not have repeat treatment as he died from unexpected stroke 4 months post-IRE. Univariable logistic regression analyses of primary technical success, complication and > 25% reduction in eGFR are summarised in Table 2.

**Table 2:** Results of univariable analyses of factors associated with primary technical success, complication and > 25% reduction in eGFR (*n*=30)

	Primary technical success		Occurrence of any complications		> 25% reduction in eGFR	
Variable and category	Odds ratio	*p* value	Odds ratio	*p* value	Odds ratio	*p* value
Age *	0.63 (0.28, 1.42)	0.27	0.74 (0.35, 1.56) 0.43		0.60 (0.26,1.39)	0.23
Sex						
Male	1	0.43	1	0.56	1	0.92
Female	2.08 (0.34, 12.7)		0.58 (0.09, 3.66)		1.11 (0.15, 7.97)	
Nephrometry score	0.95 (0.63, 1.44)	0.82	1.44 (0.91, 2.29)	0.12	1.93 (1.01, 3.71)	0.05
Grade of RCC						
1	1	1.00	1	0.59	1	0.57
2/3	1 (0.2, 5.04)		0.63 (0.11, 3.48)		0.56 (0.08, 4.01)	
Type of RCC						
Conventional	1	0.21	1	0.44	1	0.56
Others	4.31 (0.44, 41.8)		2.00 (0.34, 11.7)		0.50 (0.05, 5.24)	
Size (cm)	0.35 (0.12, 1.07)	0.07	0.51 (0.18, 1.43)	0.20	0.67 (0.22, 2.03)	0.48
</ = 2 cm	1	0.14	1	0.09	1	0.42
2.1–3 cm	0.30 (0.02, 4.06)		0.50 (0.07, 3.85)		0.33 (0.03, 4.04)	
> 3 cm	0.12 (0.01, 1.29)		0.15 (0.01, 1.68)		0.23 (0.02, 2.73)	
Length of procedure*	0.81 (0.57, 1.16)	0.25	0.90 (0.62, 1.31)	0.58	0.87 (0.57, 1.34)	0.53
Number of needles	0.64 (0.34, 1.21)	0.17	0.75 (0.37, 1.54)	0.44	0.75 (0.33, 1.71)	0.49

*Odds ratios given for a 10-unit increase in the predictor variable

There were a total of 7 complications in this cohort of patients, with an overall complication rate of 24% (95% confidence interval [CI]: 10%, 44%). Amongst them, there were Clavien-Dindo grade I (*n* = 6) and Clavien-Dindo grade III (*n* = 1). One patient developed self-limiting perinephric urine leak/hematoma immediately post-IRE; this was noted on the immediate post treatment CT scan. Five patients developed > 25% decreased in GFR in the immediate period post-IRE, requiring longer hospitalisation to monitor the renal function, without the need for immediate or long-term renal dialysis. Subsequently, 3 patients recovered to < 25% reduction in eGFR at 3–6 months post-IRE treatment. Major complication with Clavien-Dindo III was noted in one patient who developed post proximal ureteral leak/stricture and requiring long-term retrograde ureteric stenting for management and he passed away at 12 months related to cardiovascular event [14].

From all the univariable analyses, no factors were found to be significantly associated with primary technical success or complications. However, there was slight evidence that a larger tumour was associated with a lower chance of a success, but this result did not quite reach statistical significance (*p* = 0.07).

**Fig. 2:**
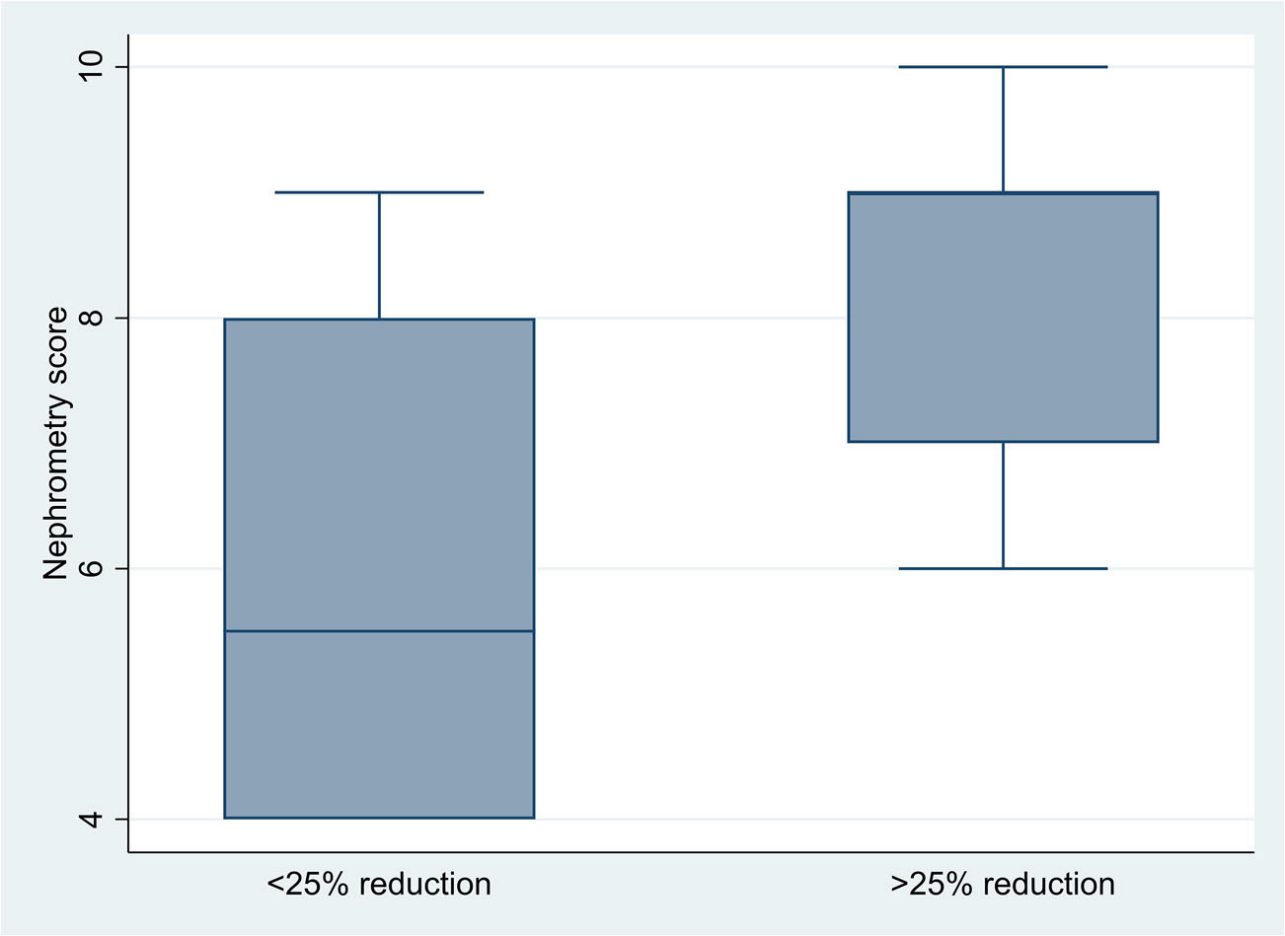
Boxplot of nephrometry score by >/< 25% eGFR reduction

There is some evidence of an association of > 25% reduction in eGFR with nephrometry score, with this result being of borderline statistical significance (*p* = 0.05). A graphical illustration of the scores for patients with and without a 25% reduction in eGFR is shown in Fig. 2. As there is only one factor that showed any association with the outcome, no further multivariable analysis had been performed.

### IRE oncological durability—LRFS, MFS, CS and OS

The initial primary technical success rate for IRE was 73% (*n* = 22/30). Local residual disease (*n* = 7) was successfully retreated with salvage CRYO without further confirmatory tumour biopsy, with an overall technical success rate of 97% (*n* = 29/30) and these patients have remained disease free on imaging for a median follow-up period of 37 months (range 12–62 months). No patients were lost to follow-up in this IRE cohort with no 30-day mortality events occurring following IRE treatment. However, there were three recorded deaths in this cohort of patients: cardiac event (*n* = 1), metastatic RCC (*n* = 1) and haemorrhagic stroke (*n* = 1).

Amongst them, two patients developed local recurrent disease in the zone of ablation at 16 and 24 months, respectively. The patient who developed local recurrent disease at 16 months (had 3.5-cm grade 3 conventional clear cell RCC) also had metastatic disease in the bone, lungs and lymph nodes and went on to have palliative systemic therapy. Unfortunately, he succumbed to metastatic RCC at 24 months after IRE. Another patient developed late local recurrent disease at 24 months (had 2.1-cm grade 2 conventional clear cell RCC) and is currently deciding whether to pursue retreatment due to the COVID-19 pandemic.

**Fig. 3:**
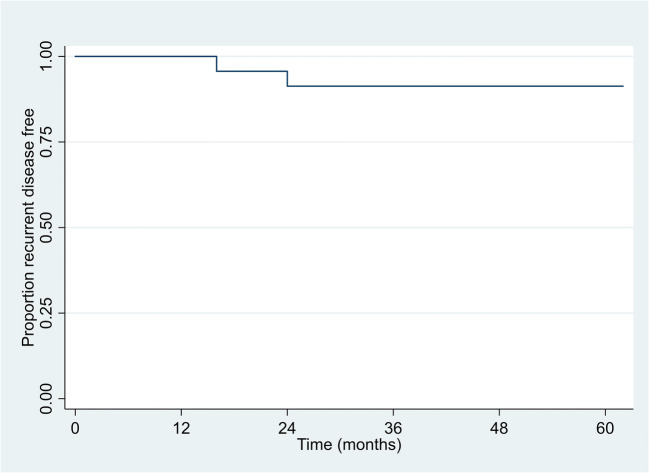
Kaplan-Meier plot of local recurrent disease-free survival (LRFS)

The 2-year and 3-year local recurrence-free survival (LRFS) rate in patients treated with IRE was 91% (95% CI: 69%, 98%) across all two time points (Fig. 3).

**Fig. 4:**
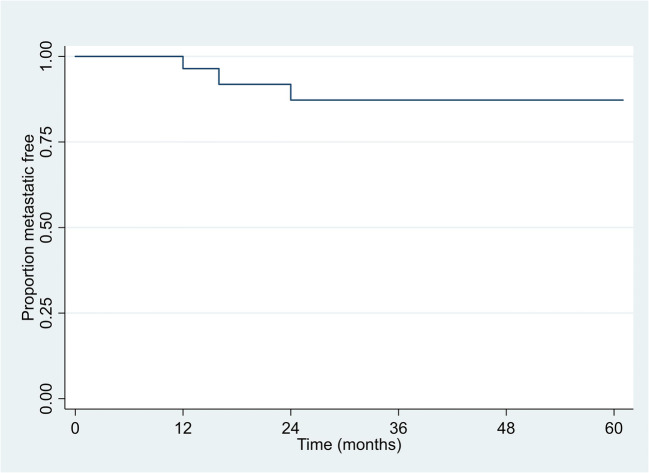
Kaplan-Meier plot of metastasis-free survival (metastatic RCC only)

A total of three patients developed metastatic disease; two were related to RCC and one patient was unrelated to RCC. Amongst them, apart from one patient with metastatic RCC who had succumbed at 24 months, the two patients with metastatic disease are still alive undergoing palliative treatment. The 2-year and 3-year metastasis-free survival (MFS) rate in patients treated with IRE and developed metastatic RCC (Fig. 4) was 87% (95% CI: 65%, 96%) across the two time points. The 2-year and 3-year cancer-specific (CS) and overall survival (OS) rates in patients treated with IRE were 96% (95% CI: 73%, 99%) and 89% (95% CI: 70%, 96%).

## Discussion

Since the first in man IRE of RCC was performed by Pech et al in 2010 [10], over the last 10 years, there are only a few (< 10) published case series on the safety and feasibility of the treatment of RCC with CT-guided IRE and a summary is tabulated (Table 3). The largest reported case series was from Canvasser et al in 2017 with the longest oncological follow-up with a mean of 22 months. The primary technical success was 93% with a 2-year actuarial LRFS and OS of 83% and 100% respectively for biopsy-proven RCC. They concluded that currently, IRE has provided suboptimal oncological durability when compared with conventional thermal ablation or partial nephrectomy [17].

**Table 3:** Summary of published case series in IRE in renal tumours (2015–2020)

	Tumours /patients/mean size (cm)	Technical success rate at 3 months	Major complication Clavien-Dindo classification (> 3)	Mean/median follow-up (mos)	2 y LRFS	2 y MFS	2 y OS
Trimmer (2015) [15]	20/20 (2.2 cm)	90% (18/20) 2 needed salvage RFA therapy	0	N/A 6 with 12 mo FU	1 at 12 mo	N/A	N/A
Diehl (2016) [16]	7/5 (2.4 cm)	100% (7/7)	0	Mean 6.4	N/A	N/A	N/A
Canvasser (2017) [17]	42/41 (2.0 cm)	93% (39/42) 3 had salvage RFA	0	Mean 22	83% (biopsy-proven/history RCC)	N/A	100% (biopsy-proven/history RCC)
Wendler (2018) [18]	7/7 (2.2 cm)	57% (4/7)	0	1	N/A	N/A	N/A
Buijs (2019) [19]	10/10 (2.2 cm)	90% (9/10)	1 (grade 3b)	Median 6	N/A	N/A	N/A
Liu (2019) [20]	5/5 (2.8 cm)	80% (4/5) had salvage RFA at 21 mo	0	Mean 22.8	1 at 21 mo	N/A	N/A
Wah (2020)	30/26 (2.5 cm)	73.3% (22/30) 7 had salvage CRYO	1 (grade 3)	Mean 36 Median 37	91%	87%	89%

Our longitudinal 5-year clinical case series has reported the longest follow-up post-IRE in RCC when compared to the current published case series with a median follow-up period of 37 months. The clinical experience has confirmed a definite learning curve for operator despite having vast experience in placing multiple needle electrodes in cryoablation (CRYO) of renal tumour (> 17 years’ experience in image-guided renal ablation) with a primary technical success rate of 73% and an overall technical success rate of 97% following salvage CRYO for 30 renal tumours. The learning curve has included the need to ensure precise positioning of the parallel needle electrode to ensure uniformity of the electrical field; reviewing the cases with local residual disease, there was evidence that some of the needle electrodes were not placed as optimally as it should be to achieve the uniformity of the voltage during IRE treatment and this was also observed by Canvasser et al, where future monitoring would be required to validate this observation [17]. Overall, the clinical outcome has confirmed the initial safety of IRE in RCC where majority of the patients had a short hospital stay (mean hospital stay of two nights) and only one Clavien-Dindo III proximal ureteric injury complication treated conservatively with retrograde ureteric stent insertion (3.4%; *n* = 1/29). The patient was a 74-year-old male with histologically confirmed type 1, grade 2 papillary centrally located left RCC abutting the left proximal ureter and underwent image-guided IRE. He developed urine leak at 1-month post-IRE during imaging follow-up and was treated with retrograde ureteric stent insertion. At 6 months post stent removal, the follow-up imaging showed permanent proximal ureteric stricture resulting in the need for permanent ureteric stent and he passed away at 12 months related to cardiovascular event [14].

It is well established that long-term oncological outcomes for cT1a RCC with 5-year LRFS for partial nephrectomy, CRYO and RFA were > 97%, 86–94% and 93–96% respectively [2, 21]. Our 5-year clinical experience has shown 2- and 3-year LRFS rate of 91% in this small cohort of patients with a median follow-up of 37 months; it is currently not as favourable as the conventional treatment. The LRFS may improve with better case selection and improved operator’s learning curve. This was also observed by Canvasser et al in their case series where the suboptimal oncological durability is 83% with LRFS at 2-year. To validate the role of image-guided IRE in the treatment of RCC, longer term follow-up with improved operator’s experience would be required to assess its oncological durability.

All the currently published clinical series are summarised in Table 3; apart from Canvasser et al [17], the rest of the studies had limited long-term follow-up for IRE in RCC. The published studies show that the technical success rate ranged from 54 to 93% with a low rate of major complications. This has confirmed that the primary technical success rate has not met the conventional treatment with surgery or image-guided CRYO or RFA of > 95% [1, 2, 4, 21, 22].

Our study has several limitations, including lack of pre-treatment biopsy in two patients, although this was on a background of recurrent RCCs with VHL disease and local residual disease was not reconfirmed at the time of retreatment with salvage CRYO. This is a small case series with retrospective analysis of a prospective collated registry and due to the relatively small number of patients, there was a low power to show associations between the factors and the outcomes. Therefore, although the results were generally not statistically significant, this may be due to the small sample size. Statistical associations may be found if data was obtained from a larger patient group.

## Conclusions

The early clinical experience showed that CT-guided IRE in small RCC is safe and with acceptable complications. Suboptimal primary technical success rate (73%) in the early operator’s learning curve and the oncological durability for 2- and 3-year LRFS, MFS, CS and OS were 91%, 87%, 96% and 89% respectively. Longer term imaging and clinical follow-up are required to validate the oncological durability of CT-guided IRE in the treatment of renal cancer.

## Abbreviations

AUA: American Urological Association
CI: Confidence interval
CS: Cancer-specific survival
CT: Computed tomography
ESMO: European Society for Medical Oncology
GFR: Glomerular filtration rate
IO: Interventional oncology
IRE: Irreversible electroporation
LRFS: Local recurrence-free survival
MDT: Multidisciplinary team
MFS: Metastasis-free survival
OS: Overall survival
RCC: Renal cell carcinoma
RCC: Renal cell carcinoma
VHL: Von Hippel Lindau
